# EEG correlates to perceived urgency elicited by vibration stimulation of the upper body

**DOI:** 10.1038/s41598-024-65289-6

**Published:** 2024-06-20

**Authors:** Wanjoo Park, Haneen Alsuradi, Mohamad Eid

**Affiliations:** 1 Engineering Division, New York University Abu Dhabi, Saadiyat Island, 129188 Abu Dhabi, United Arab Emirates; 2 Center for Artificial Intelligence and Robotics, New York University Abu Dhabi, Saadiyat Island, 129188 Abu Dhabi, United Arab Emirates; 3 Department of Electrical Engineering, New York University Abu Dhabi, Saadiyat Island, 129188 Abu Dhabi, United Arab Emirates

**Keywords:** Perception, Emotion, Sensory processing

## Abstract

Conveying information effectively while minimizing user distraction is critical to human–computer interaction. As the proliferation of audio–visual communication pushes human information processing capabilities to the limit, researchers are turning their attention to haptic interfaces. Haptic feedback has the potential to create a desirable sense of urgency that allows users to selectively focus on events/tasks or process presented information with minimal distraction or annoyance. There is a growing interest in understanding the neural mechanisms associated with haptic stimulation. In this study, we aim to investigate the EEG correlates associated with the perceived urgency elicited by vibration stimuli on the upper body using a haptic vest. A total of 31 participants enrolled in this experiment and were exposed to three conditions: no vibration pattern (NVP), urgent vibration pattern (UVP), and very urgent vibration pattern (VUVP). Through self-reporting, participants confirmed that the vibration patterns elicited significantly different levels of perceived urgency (Friedman test, Holm–Bonferroni correction, *p* < 0.01). Furthermore, neural analysis revealed that the power spectral density of the delta, theta, and alpha frequency bands in the middle central area (C1, Cz, and C2) significantly increased for the UVP and VUVP conditions as compared to the NVP condition (One-way ANOVA test, Holm–Bonferroni correction, *p* < 0.01). While the perceptual experience of haptic-induced urgency is well studied with self-reporting and behavioral evidence, this is the first effort to evaluate the neural correlates to haptic-induced urgency using EEG. Further research is warranted to identify unique correlates to the cognitive processes associated with urgency from sensory feedback correlates.

## Introduction

Notification interfaces that provide efficient and effective information communication to users without overwhelming or distracting them are critical in human–computer interaction. As the proliferation of audio–visual communication pushes human information processing capacity to its limits, researchers are turning to touch as a means of conveying information to relieve overloaded visual and auditory perceptual channels^1–3^. Recent years have seen a surge in haptic feedback research to augment or substitute visual or auditory notification methods in a wide spectrum of applications such as driver–vehicle interaction^4^, mobile phone communication^5^, interaction with visually or auditory impaired individuals^6^, and soldier–battlefield interaction^7^. Haptic feedback does not increase visual and/or auditory demands^8^, is robust to audible noise^9^, engages people at different emotional levels^10^, and is confidential^11^. In particular, vibration is becoming widely used in notification interfaces due to its low cost, wearability, and expressiveness^12^.

Vibration feedback has the potential to elicit a desirable sense of urgency in order to allow the user to selectively attend to an event/task or process the presented information with minimal distraction or annoyance^13,14^. For instance, a low urgency vibration may inform the user about the time during oral presentations without distracting them from the presentation or stopping them from going slightly over time. A high urgency vibration informs a driver that they need to pay immediate attention to avoid a collision. Existing approaches to evaluate the perceived urgency are based on self-reporting (questionnaires) and/or performance (such as response time and hit rate)^15^. While previous studies have shown that human’s self-reporting and behavioral performance are indirect indicators of cognitive state, this may not be consistent and may lead to an incorrect evaluation of the mental experience of the user^16^. Brain scanning technologies (such as fMRI, EEG, and fNIRS) enable direct evaluation of the users’ cognitive state to improve usability testing^17^. Compared to fMRI and fNIRS, EEG is the most extended technique for evaluating the user’s cognitive state as it is non-invasive, inexpensive, and fully wearable and it provides high temporal resolution^18^. Nevertheless, previous studies using EEG have primarily focused on investigating cognitive processes in response to auditory^19^ and/or visual stimulation^20^.

Urgency signaling in decision making has also been investigated using EEG^21,22^. An event-related potential (ERP)-labeled centroparietal positivity (CPP) reflects a combination of urgency and sensory evidence in decision making^23^. In order to disentangle evidence accumulation from urgency, a subsequent study revealed that the P300 (time locked to stimulus onset) represented a sensory evidence accumulation while the CPP (time locked to the choice) encoded the decision variable^24^.

A recent body of research investigated EEG neural correlates to various haptic stimuli, such as delayed kinesthetic haptic feedback^25^, presence and intensity of vibration feedback^26^, tactile texture^27^, and thermal feedback^28^. The majority of these studies have examined time-frequency features in the form of power spectral density (PSD) analysis, where the power of different frequency bands is examined for the duration of the stimulation. In particular, studies on fingertip vibration stimulation showed event-related (de)synchronization (ERD/ERS)^26^, which is a phenomenon that occurs in performing motor (such as fingers, hands, or feet) movements or imagery^29,30^; these features where exhibited in the form of power decrease/increase in the alpha and beta bands during stimulation. However, no studies have examined the EEG response to vibration stimulation of the upper body. Studies involving upper body stimulation have attempted to differentiate emotions elicited by multimodal stimulation that combines visual, auditory, and haptic inputs^31^. As such, it is difficult to distinguish whether the emotions produced were due to haptic, visual or auditory stimulation. There have also been studies that have used upper body vibration stimulation as feedback to enhance motor imagery^32^; however, these studies used upper body vibration as feedback and did not examine the EEG response to the vibration stimulus itself.

In this study, we designed two vibration patterns that are capable of eliciting two levels of urgency (urgent and very urgent) to identify EEG correlates associated with perceived urgency. While verified through self-reporting, these vibration patterns are designed by controlling the vibration parameters including the vibration intensity, duration, body part (upper body), and surface area. Given the location of the trunk in the cortical homunculus representation is at the middle central region, we hypothesize that the PSD in the middle central area significantly increases for the urgent and very urgent vibration patterns as compared to the no vibration feedback condition. Furthermore, by examining specific frequency band activation, we seek to identify neural correlates that distinguish urgent from very urgent conditions.

**Figure 1:**
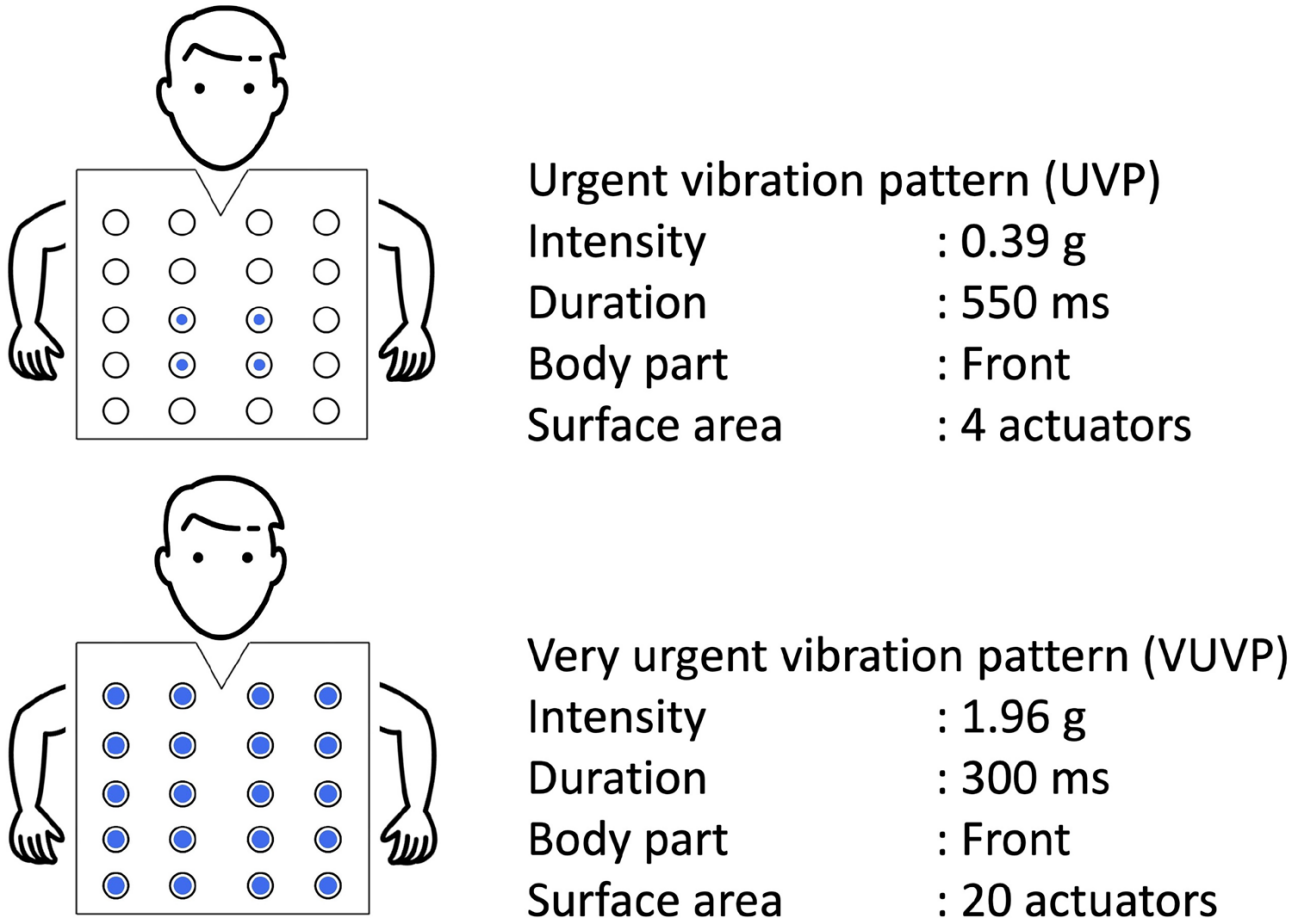
Design parameters of the employed vibration patterns. The two vibration patterns are designed to elicit two different levels of the sense of urgency. The two vibration pattern stimuli vary in intensity, duration, and area and have been developed for the upper body.

**Figure 2:**
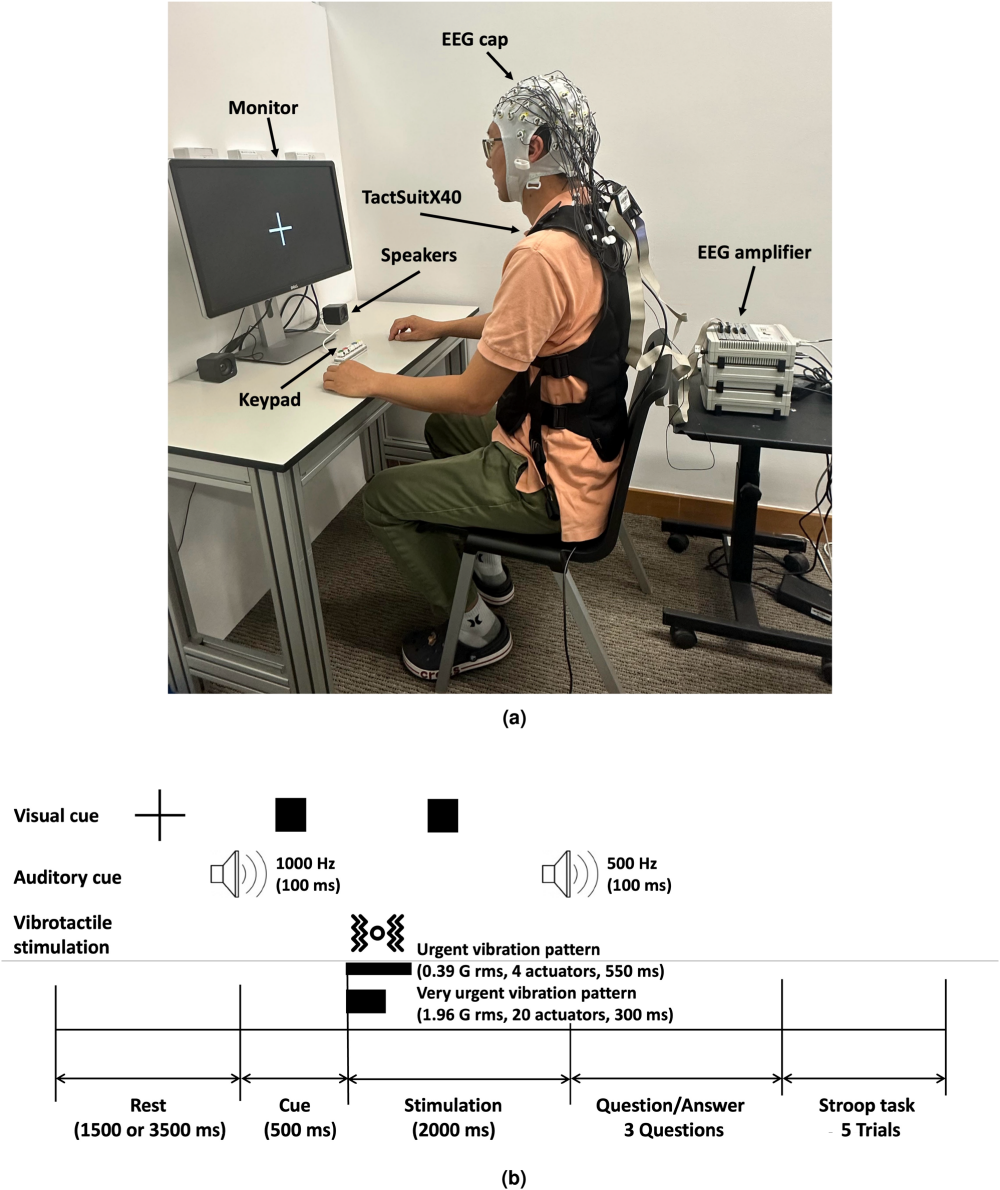
Experimental setup and protocol. (**a**) Experimental setup. Participants wore an EEG cap on the head and a haptic vest that delivers vibrotactile feedback on their upper body. (**b**) Experimental protocol. Visual and auditory cues indicate the start of the trial. The stimulation period lasts for 2000 ms, but the two vibration patterns are presented for only 550 ms and 300 ms, respectively. In the Questions/Answers period, participants are asked whether they felt any vibration and about their perceived urgency, annoyance, and acceptance.

## Materials and methods

### Participants

Thirty-one participants took part in this study. To calculate a proper number of participants, we considered intensities as an effect size with a power of 0.8. There are multiple parameters in the vibration pattern, but the intensity of the vibration is the main factor of the vibration. We also selected a significant level of 0.05 and assumed a standard deviation of 3. The calculated sample size was 31. As stated in the literature, considering healthy subjects, the number of 30 participants is considered an acceptable number^33,34^. Twenty-one of them were males and 10 of them were females, with an age range of 18–39 years old. All participants had either a normal or corrected-to-normal vision as well as a pain-free upper body. The exclusion criteria were being under 18 or having a history of traumatic brain injury, neurological disorders, or muscle atrophy. All participants were informed about the protocol of the experiment and signed the informed consent form. They also agree to publish the information/image(s) in an online open-access publication. The protocol followed in this study was approved by the New York University Abu Dhabi Institutional Review Board (HRPP-2022-96) and was in accordance with the Declaration of Helsinki, following its guidelines and regulations. All participants received monetary compensation for their participation in the study.

### Experimental setup

In this experiment, we have used a haptic vest called the TactSuitX40 (bHaptics Inc., Daejeon, South Korea) to deliver the vibrotactile stimulation to the upper body including the chest, abdomen, shoulders, and the back. The TactSuitX40 was connected wirelessly to the PC through Bluetooth communication. Participants faced a screen where the experimental sequence was presented. The experiment was designed using Psychopy (Version 2023.1.2). The bHaptics Python library was used to interface the pre-designed vibration patterns with the experimental routine.

As for EEG recording, EEG data were recorded using a BrainAmps Standard amplifier (Brain Products GmbH, Germany. https://brainvision.com/products/brainamp-standard) at a sampling frequency of 1 kHz. We have used the Brain Vision Recorder software to manage the data acquisition and monitor electrode impedance. The EEG signals were acquired by a 64-channel EEG cap (EasyCap, Herrsching, Germany) positioned according to the 10–20 international system. The ground electrode was placed at the FPz location while the online reference electrode was placed at the FCz location. To ensure high-quality recording, the impedance of each electrode was kept below 10 k$\Omega$. The experimental setup is shown in Fig. 2a.

### Experimental protocol

Participants were seated comfortably on a chair in front of a computer screen and were assisted to wear the haptic vest ensuring it was tight enough to feel the vibrations but without causing suffocation or discomfort. After the EEG cap was placed on the participant’s head and the gel was applied to ensure high conductance between the electrodes and the scalp, the experiment commenced. The experiment consisted of 13 runs, where the first was kept for training purposes. In each run, there were 9 trials in a counterbalanced order following a Latin square between three conditions: no vibration pattern (NVP), urgent vibration pattern (UVP), and very urgent vibration pattern (VUVP). Vibration patterns of UVP and VUVP are shown in Fig. 1. Each of the vibration patterns had its own duration and intensity: the UVP had a duration of 550 ms with an intensity of 0.39 g, while the VUVP had a duration of 300 ms and an intensity of 1.96 g. Furthermore, each vibration pattern employed a different number of motors located at the chest and abdomen: four actuators were used for the UVP, while twenty actuators were used for the VUVP. Those three vibration conditions (NVP, UVP, and VUVP) were designed to elicit three different levels of urgency. The timeline of a single trial is shown in Fig. 2b. The trial starts with a rest period randomized between 1500 and 3500 ms, followed by simultaneous visual (lasting for 500 ms) and auditory (lasting for 100 ms) cues to grab the participant’s attention. This is followed by the stimulation period which is of 2000 ms duration. During the stimulation period, participants either experienced no vibration at all, a UVP, or a VUVP, delivered through the haptic vest. Thus, each participant experienced 36 trials for each urgency condition. The stimulation period was followed by three questions assessing the level of urgency, annoyance, and acceptance experienced due to the delivered vibration pattern. These questions were assessed using a 5-point Likert scale. To avoid saturation due to repetitive vibration, five trials of the Stroop task^35^ for wash-off purposes were presented after every trial of stimulation, where participants had to report whether the word presented on the screen matches its color or not through a keyboard button. A single Stroop trial lasts for at least 2 s, or longer if a response is recorded after this duration. After the completion of the 13 runs, participants filled out a short post-experiment questionnaire capturing their experience. The questionnaire inquired about the perceived number of conditions during the stimulation period.

### Data analysis

Analysis of the EEG data was performed offline using MATLAB release 2022a (MathWorks, United States) and EEGLAB toolbox (v14.1.2)^36^. The 64-channel Easycap from Brain Products was used to collect the EEG signal. However, only 60 channels were considered excluding FT9, FT10, TP9, and TP10. These four channels are located outside the brain’s area, near the ears. EEG data were band-pass filtered between 0.1 and 85 Hz using a Hamming windowed since FIR filter, followed by a notch filter at 50 Hz to suppress power line noise. For artifact removal and data cleaning, the artifact subspace reconstruction method was applied^37^. A channel was flagged if it exhibited more than five seconds of flat line, line noise exceeding four standard deviations relative to its signal, or a correlation with its reconstructed version based on other channels below 0.85. The spherical method of channel interpolation was used to mitigate the impact of these channels^38^. Furthermore, the common average referencing method was used to re-reference the EEG data. Subsequently, EEG data were epoched such that 3000 ms before and 4000 ms after the vibration onset were extracted. Epoched EEG data in time-domain were baseline corrected using a baseline between − 1000 and − 500 ms before the onset through a simple subtraction.

After pre-processing, EEG data were converted to the time–frequency domain using Morlet Wavelet transformation with 1-cycle wavelet. The frequency range considered is between 1 and 80 Hz. After obtaining the power of the transformed epochs, trials of the same condition were averaged for each participant. This is followed by decibel conversion and baseline normalization by subtraction using a baseline window from − 1000 and − 500 ms with respect to the onset. Both the cue and stimulation periods were normalized with respect to the baseline. Finally, the power of delta (1–4 Hz), theta (4–7 Hz), alpha (8–12 Hz), beta (13–30 Hz), gamma (31–50 Hz), and high gamma (51–80 Hz) band were extracted.

### Statistical analysis

As for the statistical analysis of the EEG data, it was performed on the time-frequency representation of the data, where the difference between the three urgency conditions was assessed for each frequency band, channel, and time point. We have considered the 2000 ms of stimulation, where the resolution of each time-bin after the Morlet wavelet transformation is 29 ms. The normality of the activation for each time-bin is assessed using the Jarque–Bera test; if normality is confirmed, the One-way ANOVA test is employed, otherwise, the Kruskal–Wallis test is applied. The Holm–Bonferroni correction method was used to correct the *p*-values across the three conditions as there were multiple comparisons among the three conditions with the same hypothesis. On the other hand, there were multiple comparisons with different test hypotheses depending on different channels, time points, and frequency bands. Thus, the Benjamini and Hochberg false discovery rate (FDR) test was used to correct the *p*-values from the multiple comparisons.

We also analyzed the self-reported data, which included ratings for perceived urgency, annoyance, and acceptance of each trial, using the Friedman test due to the non-normal distribution of ratings and repeated measures data. To account for multiple comparisons across the three conditions, we applied the Holm–Bonferroni correction. Pearson’s linear correlation was also used to examine the relationships between participants’ responses/EEG responses and the NVP, UVP, and VUVP conditions.

**Figure 3:**
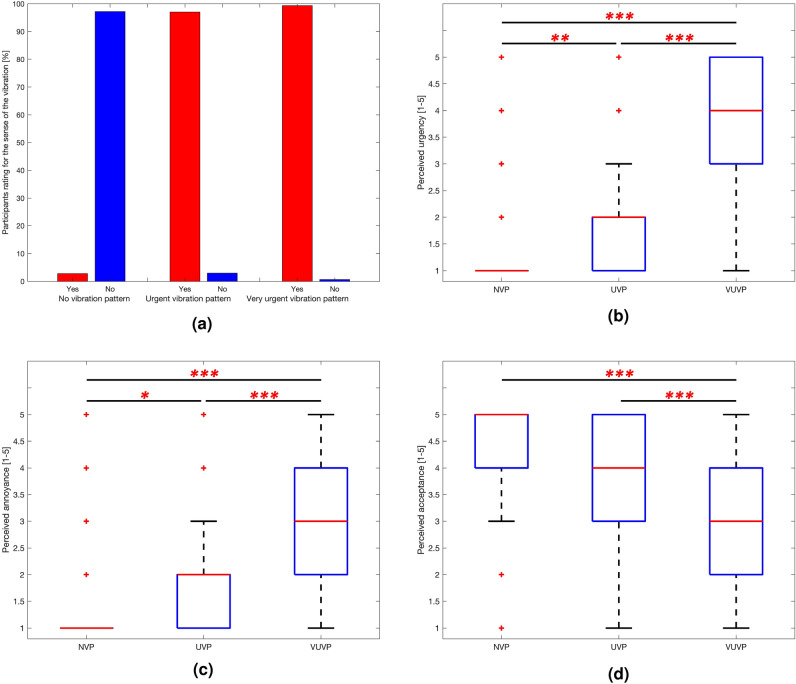
Participants’ perceived sensation. These results show the participants’ responses to the perceived sensation from the Questions/Answers period. (**a**) Perceived vibration. Whether or not participants detected the vibration.(**b**) Perceived urgency. The box plot shows the distribution of participants’ perceived urgency on a 5-point Likert scale across stimulus conditions. Friedman test, Holm-Bonferroni correction, ∗∗p < 0.01, ∗∗∗p < 0.001.(**c**) Perceived annoyance. The box plot shows the distribution of participants’ perceived annoyance on a 5-point Likert scale across stimulus conditions. Friedman test, Holm-Bonferroni correction, ∗p < 0.05, ∗∗∗p < 0.001.(**d**) Perceived acceptance. The box plot shows the distribution of participants’ perceived acceptance on a 5-point Likert scale across stimulus conditions. Friedman test, Holm-Bonferroni correction, ∗∗∗p < 0.001.

**Figure 4:**
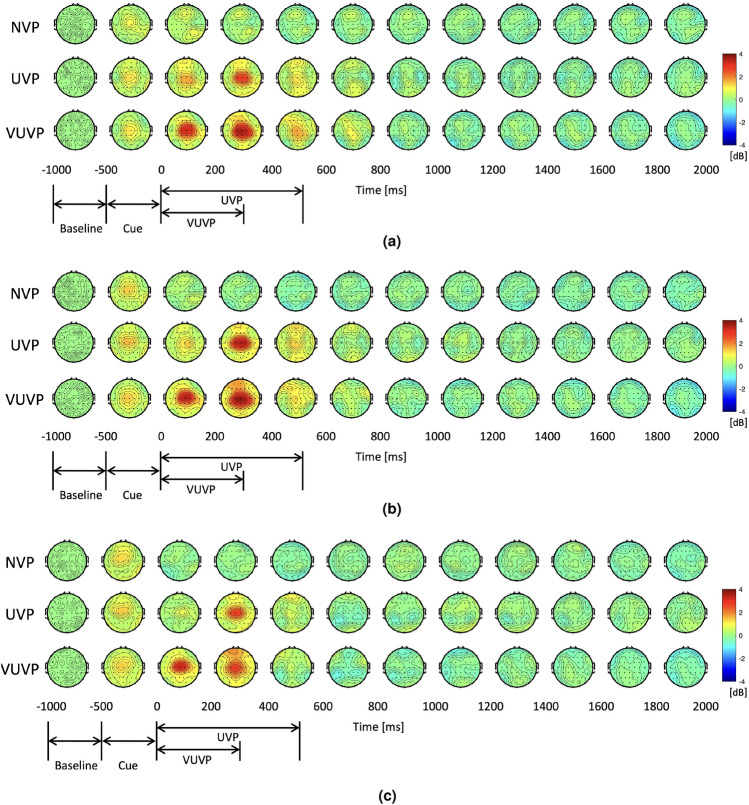
Topography analysis. NVP, UVP, and VUVP indicate no vibration pattern, urgent vibration pattern, and very urgent vibration pattern respectively. The cue period indicates the time when visual and auditory cues appeared. The color of the topography indicates the PSD changes compared to the baseline. (**a**) Topography maps of the delta band. (**b**) Topography maps of the theta band. (**c**) Topography maps of the alpha band.

## Results

### Subjective evaluation of perceived urgency

Participants answered questions after each vibration pattern stimulus. Figure 3 shows the participants perceived level of urgency, annoyance, and acceptance as well as recognition of the vibration. Participants reported that they felt no vibration for NVP stimulus with 97.23% accuracy and felt a vibration for UVP and VUVP stimuli with 97.04% and 99.35% accuracy respectively (Fig. 3a). This allowed us to verify that the experiment was conducted properly by checking for the presence or absence of urgent vibrations felt by the participants.

As for the second question, participants were asked about their perceived urgency. The results in Fig. 3b show that the level of perceived urgency of the UVP stimulus was significantly higher than the perceived urgency of the NVP stimulus (Repeated measures nonparametric one-way ANOVA, Friedman test, Holm–Bonferroni correction, *p* < 0.01), and the level of perceived urgency of the VUVP stimulus was significantly higher than the perceived urgency of the UVP stimulus (Repeated measures nonparametric one-way ANOVA, Friedman test, Holm–Bonferroni correction, *p* < 0.001). This confirms that the stimuli used to create the sense of urgency actually caused the participants to experience different levels of perceived urgency.

The third question was about perceived annoyance. Figure 3c shows participants’ responses to perceived annoyance for the NVP, UVP, and VUVP conditions. The level of perceived annoyance of the UVP stimulus was significantly higher than the perceived annoyance of the NVP stimulus (Repeated measures nonparametric one-way ANOVA, Friedman test, Holm–Bonferroni correction, *p* < 0.05), and the level of perceived annoyance of the VUVP stimulus was significantly higher than the perceived annoyance of the UVP stimulus (Repeated measures nonparametric one-way ANOVA, Friedman test, Holm–Bonferroni correction, *p* < 0.001).

The last question was about perceived acceptance. Figure 3d shows participants’ responses to perceived acceptance of the NVP, UVP, and VUVP conditions. There was no significant difference between the participants’ acceptance ratings for the NVP and UVP stimuli. However, the level of perceived acceptance of the VUVP stimulus was significantly lower than the perceived urgency of the NVP and UVP stimuli (Repeated measures nonparametric one-way ANOVA, Friedman test, Holm–Bonferroni correction, *p* < 0.001). Pearson’s linear correlation was used to examine whether participants’ responses to perceived urgency, annoyance, and acceptance were correlated with NVP, UVP, and VUVP. The results showed that there is a strong correlation with Pearson’s linear correlation coefficient, 0.7541 (*p* < 0.001) for perceived urgency. Also, there are correlations with Pearson’s linear correlation coefficient, 0.5781 (*p* < 0.001) and − 0.3348 (*p* < 0.001) for annoyance and acceptance.

From the post-experiment questionnaire, we also investigated how many conditions were recognized by the participants. Even though we provided three conditions of vibration patterns, 39% of the participants answered that they recognized four conditions. Only 29% of the participants answered that they recognized three conditions and 22% of the participants answered that they recognized five conditions. As many as 10 % of the participants answered that they recognized six conditions.

**Figure 5:**
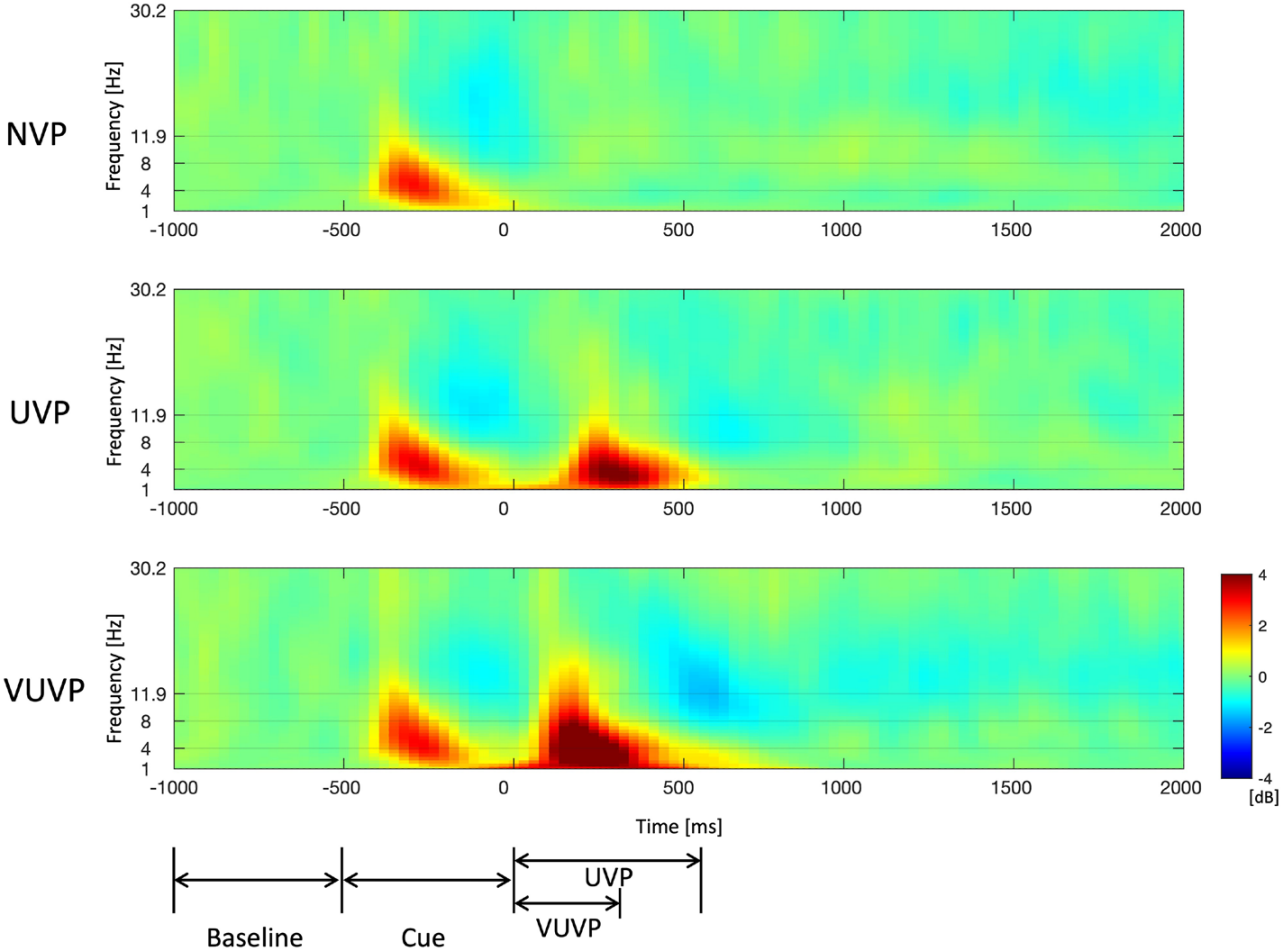
Spectrogram analysis in the middle central area (C1, Cz, and C2). NVP, UVP, and VUVP refer to no vibration pattern, urgent vibration pattern, and very urgent vibration pattern, respectively. The cue period indicates the time when visual and auditory cues appeared. The color of the topography indicates the PSD changes compared to the baseline.

### EEG-based neural analysis

#### Topography and spectrogram analysis

Topographic plots were computed for the delta, theta, alpha, beta, and gamma frequency bands for each urgency condition. Figure 4a shows the topography maps of the delta frequency band. Prior to the onset (− 500 to 0 ms), similar delta activation is observed across conditions, given that participants experienced the same auditory/visual instruction cues. However, after the onset, delta activity visibly increased for the UVP and VUVP conditions as compared to the NVP condition up to 600 ms and was largely regionalized to the middle central area. Even though the VUVP stimulus is shorter in duration than the UVP stimulus, delta power by the VUVP stimulus in the middle central area appeared earlier and was sustained longer than delta power in the UVP condition. After 1000 ms, there were no significant differences in the delta power among the NVP, UVP, and VUVP conditions. Theta and alpha activities demonstrated similar patterns as in the delta frequency band, as shown in Fig. 4b,c, respectively. These results indicate activation in the primary somatosensory brain region (known as the sensory homunculus) referring to the chest body part where the vibration stimulation is applied. No significant differences were observed in the beta and gamma bands.

Consequently, the middle central area (C1, Cz, and C2) was selected as a region of interest (ROI) for the frequency band 1–11.9 Hz (delta, theta, and alpha bands). Figure 5 shows the spectrogram of the average power in the middle central area. Given participants experienced the same visual/auditory cues for the three conditions, there were similar activations for the time interval between − 500 and 0 ms. However, there was a significant increase in the average power from the onset up to 500 ms in the VUP and VUVP conditions, as compared to the NVP condition. In the UVP condition, the delta and theta power significantly increased after around 150 ms from the onset. The spectrogram shows that the increase in delta power is more sustained than the increase in theta power. In the VUVP condition, the delta, theta, and alpha power increased earlier than in the UVP condition. Also, the increase in delta, theta, and alpha power in the VUVP condition is greater than that of the UVP condition. For the VUVP condition, a decrease in alpha and low beta power was observed between approximately 500 ms and 700 ms. After 1000 ms, there were no visible differences in the delta, theta, alpha, or beta power between the NVP, UVP, and VUVP conditions. Other frequency bands were also investigated, however, there were no significant power changes in the middle central area in the gamma and high gamma frequency bands.

#### Time course PSD analysis

**Figure 6:**
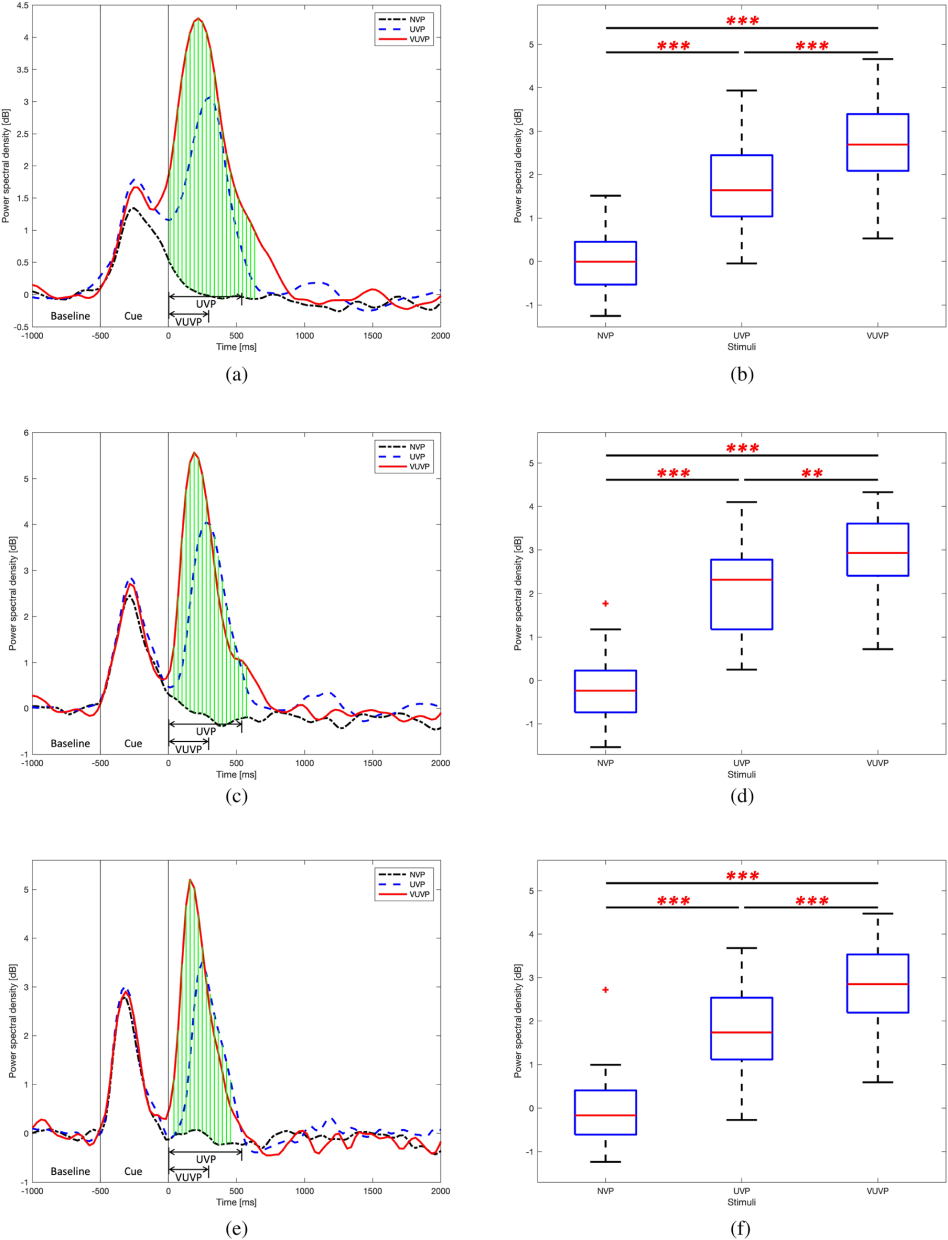
(**a,c,e**) Time course of power spectral densities in the middle central area (C1, Cz, and C2). NVP, UVP, and VUVP indicate no vibration pattern, urgent vibration pattern, and very urgent vibration pattern respectively. The cue period indicates the time when visual and auditory cues appeared. The vertical green lines mark the time-bins where the PSDs are statistically significant across three vibration conditions. One-way ANOVA test or Kruskal–Wallis test, Benjamini and Hochberg false discovery rate correction, *p* < 0.01. (**b,d,f**). The mean was taken for the time window that showed statistically significant differences among three vibration conditions for each of the bands. One-way ANOVA test, Holm–Bonferroni correction, ^∗∗^p < 0.01, ^∗∗∗^p < 0.001. (**a**) Time course of delta PSD. (**b**) Mean delta PSD from the onset to 630 ms. (**c**) Time course of theta PSD. (**d**) Mean theta PSD from the onset to 600 ms. (**e**) Time course of alpha PSD. (**f**) Mean alpha PSD from the onset to 455 ms.

The time course of the PSD analysis examined how the delta, theta, and alpha activities in the middle central area (C1, Cz, and C2) varied over time. Figure 6a shows the time course of delta PSD. As with the topography and spectrogram results, the delta PSD increased due to the auditory and visual cues prior to the onset (− 500 to 0 ms) in a similar manner for all three urgency conditions. After the onset, no delta PSD activation was observed in the NVP condition since there was no vibration stimulation. It can be seen that the activation of delta PSD in the VUVP condition appeared sooner than that in the UVP condition, even though the UVP and the VUVP stimuli started at the same time (the onset). Furthermore, the delta PSD peak was higher for the VUVP condition than that for the UVP condition. As shown in (Fig. 6a), significant differences in the delta PSD between the three conditions were found from 0 to 630 ms where vertical green lines show the time-bins of significant differences between the three conditions (one-way ANOVA or Kruskal–Wallis test, Benjamini and Hochberg false discovery rate correction, *p* < 0.01). Figure 6b shows that the mean delta PSD for the VUVP condition was significantly higher than that of the UVP condition, which in turn was significantly higher than that in the NVP condition (one-way ANOVA test, Holm–Bonferroni correction, *p* < 0.001).

A similar activation pattern was observed for theta PSD. Figure 6c shows the time course of the theta PSD, where the time-bins showing significant differences between the three conditions are highlighted (0-600 ms) (One-way ANOVA or Kruskal–Wallis test, Benjamini and Hochberg false discovery rate correction). In the theta band, the power in the VUVP condition was prominently enhanced compared with the UVP condition while the power in the NVP condition demonstrated negligible activation. Compared to the time course of delta PSD, the time interval for significant differences was shorter 0-600 ms (One-way ANOVA or Kruskal–Wallis test, Benjamini and Hochberg false discovery rate correction). Figure 6d shows that the mean theta PSD for the VUVP condition was significantly higher than that of the UVP condition, which was significantly higher than that of the NVP condition, for the duration 0-600 ms (one-way ANOVA test, Holm–Bonferroni correction, *p* < 0.001).

The time course of the alpha PSD also demonstrated significant differences between the three urgency conditions between 0 and 455 ms time interval, as shown in Fig. 6e (One-way ANOVA or Kruskal-Wallis test, Benjamini and Hochberg false discovery rate correction). Figure 6f shows that the mean alpha PSD for the VUVP condition was significantly higher than that of the UVP condition, which was in turn significantly higher than that of the NVP condition (one-way ANOVA test, Holm–Bonferroni correction, *p* < 0.001). Pearson’s linear correlation was used to examine whether the mean delta, theta, and alpha PSDs of the significant time windows were correlated with NVP, UVP, and VUVP. The results showed that there are strong correlations with Pearson’s linear correlation coefficients of 0.7604, 0.7961, and 0.7845 for mean delta, theta, and alpha PSDs, respectively. For these three correlation tests, *p* < 0.001.

## Discussion

The present study aims to investigate the EEG neuro-representation of perceived urgency elicited via vibration stimulation on the upper body. Given how the perceived urgency can be largely modulated by context^39^, the urgency stimulation is considered only vibration feedback without any visual or auditory context. Therefore, the EEG correlates under study are associated with the sense of urgency elicited exclusively by vibration stimulation. Examining the correlation results of the perceived urgency, annoyance, and acceptance ratings and the mean delta, theta, and alpha PSDs from the time windows of interest (i.e. significant time windows), it is clear that self-reporting and neural correlates are well correlated for the NVP, UVP, and VUVP conditions.

While our results reveal significant differences in delta, theta, and alpha frequency bands across the three urgency conditions, it is not easy to distinguish whether these differences are due to the sensory experience of different vibration patterns or due to cognitive processes associated with urgency elicited by the vibration stimulation. From the topography results in Fig. 4, it is shown that the stimulus-induced response is in the middle central region. This is the area where the somatosensory cortex of the chest and abdomen are located^40^. Thus, the EEG activation due to the vibration stimulation could be a sensory response. However, in a previous study^26^ on the EEG response to vibration stimulation on the index fingertip, the neural response commenced at the same time regardless of the intensity of the vibration stimulation. On the contrary, the results of this study show that the EEG response to VUVP is stronger and is activated earlier than the EEG response to UVP after the onset. Furthermore, the neural activation due to the VUVP is sustained longer than the UVP, with respect to their stimulation period. This suggests that the difference is not only due to the sensory experience but also due to the cognitive function of urgency. Furthermore, studies have shown that the delta^41^ and theta^42^ bands are strongly associated with cognitive functions. In addition, the alpha band is known to be strongly associated with attention^43^. The urgency elicited by the vibration pattern likely made the participants feel more alert and focused, prompting them to be ready to take action. This increase in power in the lower frequency bands can be attributed to cognitive function rather than sensation. Previous studies on the EEG response to vibration stimulation have reported a decrease in alpha and beta power in response to vibration stimulation^26^. Another interesting finding was that the alpha and low beta powers decreased between approximately 500 and 700 ms, which was only observed under the VUVP condition. Studies have shown that a decrease in alpha and beta power occurs when tracking stimulus-specific information^44^ and when activating long-term memory^45^. This is likely due to the fact that VUVPs are characterized by a clear sense of urgency associated with cognitive activity. Therefore, it can be assumed that the increase in power in these frequency bands is a neural representation of the elicited sense of urgency in addition to a sensory response to vibration stimulation. Our findings confirmed the hypothesis that neural representations differ between the no-vibration condition and the conditions of urgent and very urgent vibration patterns. These differences were evident in the delta, theta, and alpha frequency bands in the middle central area.

As for the type of activation observed, it is well known that ERD/ERS occurs when performing motor movements or imagery^29,30^. ERD/ERS is also shown in studies that involve vibration stimulation of the fingertips^26^. However, in this study, vibration stimulation was applied to the upper body where no desynchronization in power was observed. Previous research has employed vibration stimulation at the chest during emotional visual stimulation; however, there was no reference to an independent analysis of the neural response to upper-body stimulation^31^. Additionally, another study used the same haptic vest as in this research as a feedback modality during motor imagery. In this case, too, no EEG analysis was presented, with the focus being solely on motor imagery classification^32^.

A few limitations of the current study are also acknowledged. First, urgency is considered in isolation (based solely on vibration feedback). Future research should consider the effects of context (visual and/or auditory interaction) on the perceived urgency (e.g. driving context). Secondly, the current study considered one age group (18–39 years old) and therefore other age groups should also be considered. Finally, given how studies on upper body stimulation are very limited, further research is warranted to better understand the EEG correlates to vibration stimulation of the upper body in general.

### Supplementary Information

Supplementary Information.

## Data Availability

Datasets generated and/or analyzed during this study are available upon request from corresponding authors.

## References

[1] Ahtamad M, Spence C, Ho C, Gray R (2015). Warning drivers about impending collisions using vibrotactile flow. IEEE Trans. Haptics.

[2] Di Campli San Vito, P. *et al.* Purring wheel: Thermal and vibrotactile notifications on the steering wheel. In *Proc. 2020 International Conference on Multimodal Interaction* 461–469 (2020).

[3] Lutnyk L, Rudi D, Meier E, Kiefer P, Raubal M (2022). Flybrate. Evaluating vibrotactile cues for simulated flight. Int. J. Hum. Comput. Interact..

[4] Chiossi, F. & Chuang, L.L. Notification in automation: Haptic feedback for supporting safety in automated driving. In *AutomationXP@ CHI* (2020).

[5] Sim J, Yim Y, Kim K (2019). Development and evaluation of the haptiwatch with a smart notification system. Hum. Fact. Ergon. Manuf. Serv. Ind..

[6] Furuhashi, M., Nakamura, T., Kanoh, M. & Yamada, K. Haptic communication robot for urgent notification of hearing-impaired people. In *2016 11th ACM/IEEE International Conference on Human–Robot Interaction (HRI)* 429–430 (IEEE, 2016).

[7] White TL (2011). The Perceived Urgency of Tactile Patterns.

[8] Shah, V. A. *et al.* Effect of dual tasking on vibrotactile feedback guided reaching—A pilot study. In *International Conference on Human Haptic Sensing and Touch Enabled Computer Applications* 3–14 (Springer, 2018). 10.1007/978-3-319-93445-7_1 PMC6555617 31179445

[9] Azenkot, S. *et al.* Enhancing independence and safety for blind and deaf-blind public transit riders. In *Proc. SIGCHI Conference on Human Factors in Computing Systems* 3247–3256 (2011).

[10] Réhman, S. U. & Liu, L. ifeeling: Vibrotactile rendering of human emotions on mobile phones. *Mobile Multimedia Processing: Fundamentals, Methods, and Applications* 1–20 (2010).

[11] Ferris TK, Sarter N (2011). Continuously informing vibrotactile displays in support of attention management and multitasking in anesthesiology. Hum. Fact..

[12] Papetti, S., Fröhlich, M., Fontana, F., Schiesser, S. & Avanzini, F. Implementation and characterization of vibrotactile interfaces. In *Musical Haptics* 257–282 (Springer, 2018).

[13] MacLean KE (2009). Putting haptics into the ambience. IEEE Trans. Haptics.

[14] Asplund CL, Obana T, Bhatnagar P, Koh XQ, Perrault ST (2020). It’s all in the timing: Principles of transient distraction illustrated with vibrotactile tasks. ACM Trans. Comput. Hum. Interact..

[15] Burt, J. L., Bartolome-Rull, D. S., Burdette, D. W. & Comstock, J. R. A psychophysiological evaluation of the perceived urgency of auditory warning signals. In *Human Factors in Auditory Warnings* 151–170 (Routledge, 2019). 10.1080/00140139508925271 7498191

[16] Peck, E. M. M., Yuksel, B. F., Ottley, A., Jacob, R. J. & Chang, R. Using fnirs brain sensing to evaluate information visualization interfaces. In *Proc. SIGCHI Conference on Human Factors in Computing Systems* 473–482 (2013).

[17] Hall PA, Burhan AM, McKillop JC, Duarte D (2023). Next-generation cognitive assessment: Combining functional brain imaging, system perturbations and novel equipment interfaces. Brain Res. Bull..

[18] Dvorak D, Shang A, Abdel-Baki S, Suzuki W, Fenton AA (2018). Cognitive behavior classification from scalp eeg signals. IEEE Trans. Neural Syst. Rehabil. Eng..

[19] Lee, Y.-C. *et al.* An eeg-based approach for evaluating audio notifications under ambient sounds. In *Proc. SIGCHI Conference on Human Factors in Computing Systems* 3817–3826 (2014).

[20] Vi, C. & Subramanian, S. Detecting error-related negativity for interaction design. In *Proc. SIGCHI Conference on Human Factors in Computing Systems* 493–502 (2012).

[21] Thura D, Beauregard-Racine J, Fradet C-W, Cisek P (2012). Decision making by urgency gating: Theory and experimental support. J. Neurophysiol..

[22] Yau Y (2020). Neural correlates of evidence and urgency during human perceptual decision-making in dynamically changing conditions. Cereb. Cortex.

[23] O’connell RG, Dockree PM, Kelly SP (2012). A supramodal accumulation-to-bound signal that determines perceptual decisions in humans. Nat. Neurosci..

[24] Yau Y (2021). Evidence and urgency related eeg signals during dynamic decision-making in humans. J. Neurosci..

[25] Alsuradi H, Park W, Eid M (2021). Midfrontal theta oscillation encodes haptic delay. Sci. Rep..

[26] Park W, Kim S-P, Eid M (2021). Neural coding of vibration intensity. Front. Neurosci..

[27] Moungou A, Thonnard J-L, Mouraux A (2016). Eeg frequency tagging to explore the cortical activity related to the tactile exploration of natural textures. Sci. Rep..

[28] Park W, Korres G, Jamil MH, Eid M (2023). Neural correlates of thermal stimulation during active touch. Front. Neurosci..

[29] Neuper C, Wörtz M, Pfurtscheller G (2006). Erd/ers patterns reflecting sensorimotor activation and deactivation. Prog. Brain Res..

[30] Pfurtscheller G (2001). Functional brain imaging based on erd/ers. Vis. Res..

[31] Li D (2022). Eeg-based emotion recognition with haptic vibration by a feature fusion method. IEEE Trans. Instrum. Meas..

[32] Arpaia P (2023). Visual and haptic feedback in detecting motor imagery within a wearable brain–computer interface. Measurement.

[33] Alsuradi H, Park W, Eid M (2022). Midfrontal theta power encodes the value of haptic delay. Sci. Rep..

[34] Rodionov A (2023). Reliability of resting-state eeg modulation by continuous and intermittent theta burst stimulation of the primary motor cortex: A sham-controlled study. Sci. Rep..

[35] Logan GD, Zbrodoff NJ, Williamson J (1984). Strategies in the color-word stroop task. Bull. Psychon. Soc..

[36] Delorme A, Makeig S (2004). Eeglab: An open source toolbox for analysis of single-trial eeg dynamics including independent component analysis. J. Neurosci. Methods.

[37] Kothe, C. A. E. & Jung, T.-P. *Artifact Removal Techniques with Signal Reconstruction. US Patent App. 14/895,440* (2016).

[38] Delorme A (2023). Eeg is better left alone. Sci. Rep..

[39] Maximini D, Maximini D, Vinaja R (2015). Creating a sense of urgency. The Scrum Culture: Introducing Agile Methods in Organizations.

[40] Purves D (2013). Principles of Cognitive Neuroscience.

[41] Harmony T (2013). The functional significance of delta oscillations in cognitive processing. Front. Integr. Neurosci..

[42] Soltani Zangbar H (2020). Theta oscillations through hippocampal/prefrontal pathway: Importance in cognitive performances. Brain Connect..

[43] Klimesch W, Doppelmayr M, Russegger H, Pachinger T, Schwaiger J (1998). Induced alpha band power changes in the human eeg and attention. Neurosci. Lett..

[44] Griffiths BJ (2019). Alpha/beta power decreases track the fidelity of stimulus-specific information. eLife.

[45] Hanslmayr S, Staudigl T, Fellner M-C (2012). Oscillatory power decreases and long-term memory: The information via desynchronization hypothesis. Front. Hum. Neurosci..

