# Supplementary material for: EEG correlates to perceived urgency elicited by vibration stimulation of the upper body
Source: Sci Rep. 2024 Jun 20;14:14267. doi: 10.1038/s41598-024-65289-6 (PMC11189896; doi:10.1038/s41598-024-65289-6)
Supplement: Supplementary file 1 — Supplementary Information. [file 41598_2024_65289_MOESM1_ESM.pdf]

Supplementary Materials

Variables	Statistical tests	*p-values*	Figures
Perceived urgency	Friedman test, Holm-Bonferroni correction	$p < 0.01$	Figure 3(b)
Perceived annoyance	Friedman test, Holm-Bonferroni correction	$p < 0.05$	Figure 3(c)
Perceived acceptance	Friedman test, Holm-Bonferroni correction	$p < 0.001$	Figure 3(d)
Perceived urgency	Pearson's linear correlation	$p < 0.001$	Figure 8
Perceived annoyance	Pearson's linear correlation	$p < 0.001$	Figure 9
Perceived acceptance	Pearson's linear correlation	$p < 0.001$	Figure 10
Time course of delta PSD	One-way ANOVA test or Kruskal-Wallis test, BH FDR correction	$p < 0.01$	Figure 6(a)
Time course of theta PSD	One-way ANOVA test or Kruskal-Wallis test, BH FDR correction	$p < 0.01$	Figure 6(c)
Time course of alpha PSD	One-way ANOVA test or Kruskal-Wallis test, BH FDR correction	$p < 0.01$	Figure 6(e)
Mean delta PSD	One-way ANOVA test, Holm-Bonferroni correction	$p < 0.001$	Figure 6(b)
Mean theta PSD	One-way ANOVA test, Holm-Bonferroni correction	$p < 0.01$	Figure 6(d)
Mean alpha PSD	One-way ANOVA test, Holm-Bonferroni correction	$p < 0.001$	Figure 6(f)
Mean delta PSD	Pearson's linear correlation	$p < 0.001$	Figure 10
Mean theta PSD	Pearson's linear correlation	$p < 0.001$	Figure 11
Mean alpha PSD	Pearson's linear correlation	$p < 0.001$	Figure 12

Supplementary Table 1. Statistical tests and results. BH FDR indicates Benjamini and Hochberg false discovery rate.

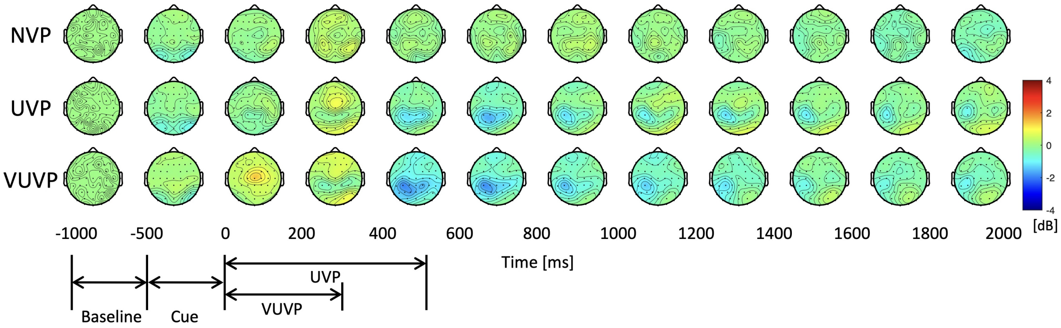

Supplementary Figure 1. Topography maps of the beta band. NVP, UVP, and VUVP indicate no vibration pattern, urgent vibration pattern, and very urgent vibration pattern, respectively. The cue period indicates the time when visual and auditory cues appeared. The color of the topography indicates the PSD changes compared to the baseline.

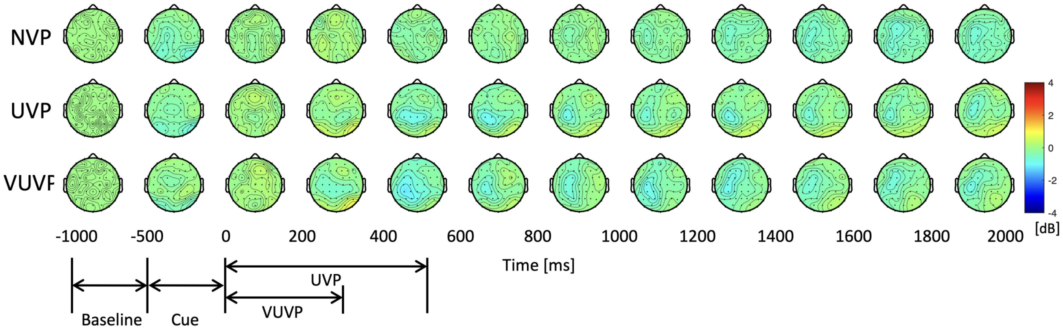

Supplementary Figure 2. Topography maps of the gamma band. NVP, UVP, and VUVP indicate no vibration pattern, urgent vibration pattern, and very urgent vibration pattern, respectively. The cue period indicates the time when visual and auditory cues appeared. The color of the topography indicates the PSD changes compared to the baseline.

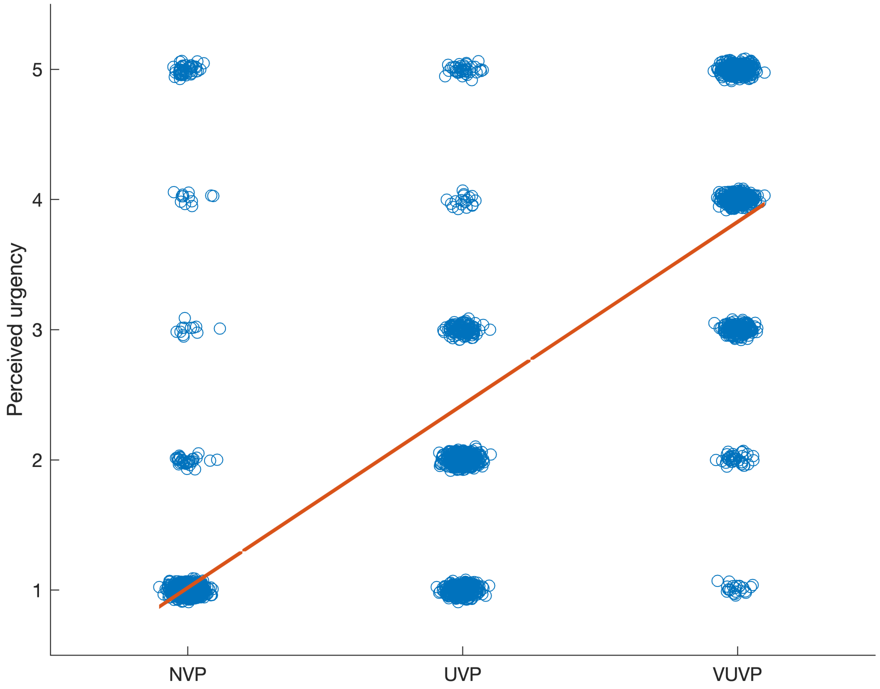

Supplementary Figure 3. Participants' responses to perceived urgency for the NVP, UVP, and VUVP conditions. Pearson's linear correlation coefficient, 0.7541, $p < 0.001$.

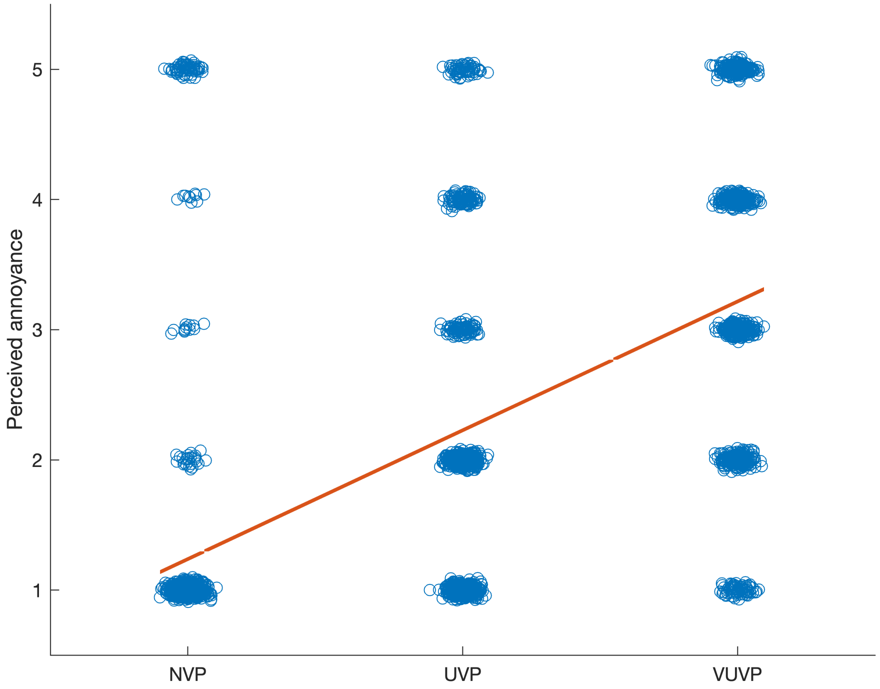

Supplementary Figure 4. Participants' responses to perceived annoyance for the NVP, UVP, and VUVP conditions. Pearson's linear correlation coefficient, 0.5781, $p < 0.001$.

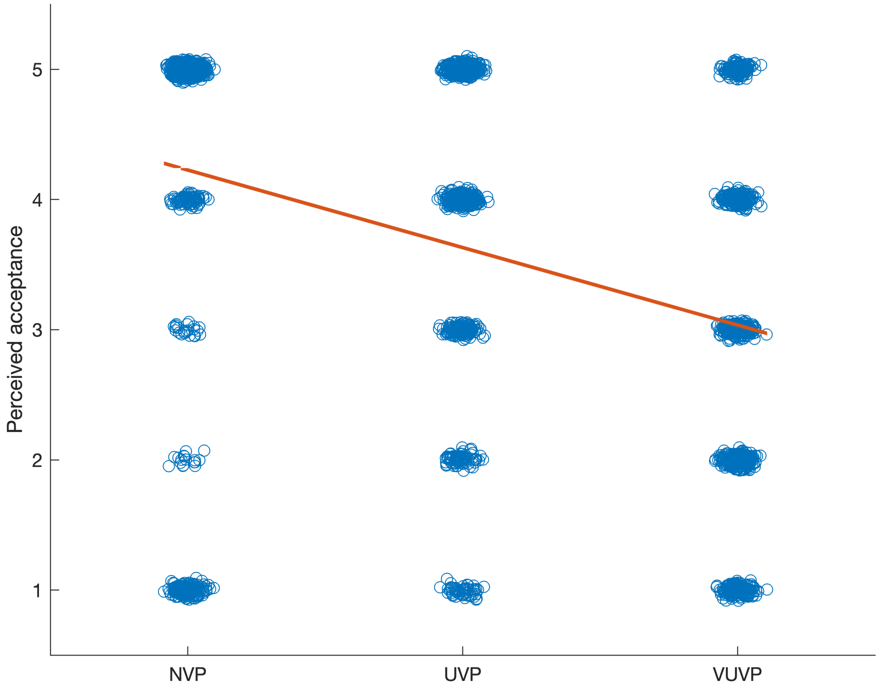

Supplementary Figure 5. Participants' responses to perceived acceptance for the NVP, UVP, and VUVP conditions. Pearson's linear correlation coefficient, -0.3348, $p < 0.001$.

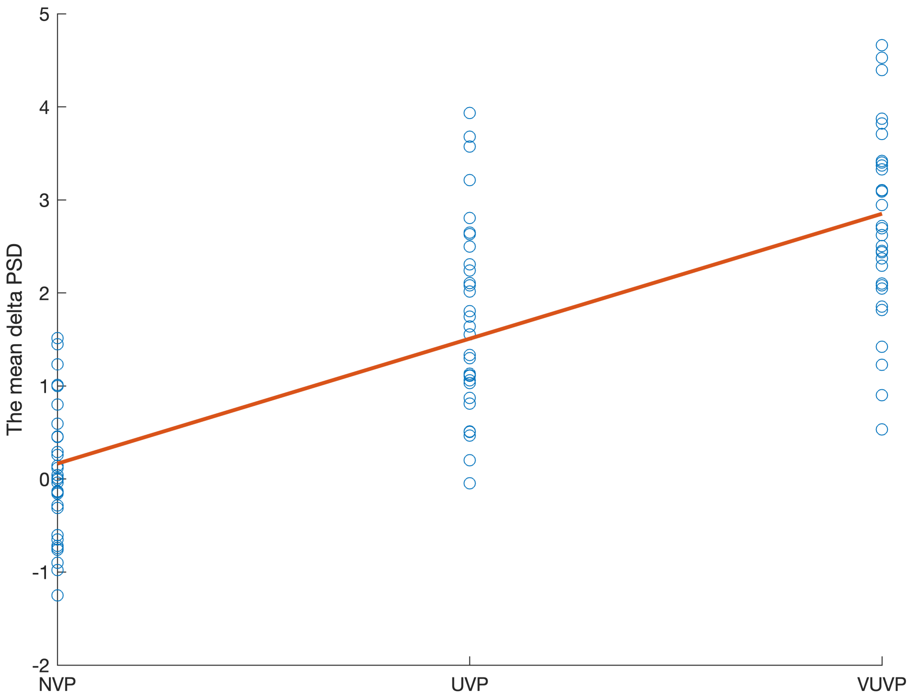

Supplementary Figure 6. The mean delta PSD within the significant time window for the NVP, UVP, and VUVP conditions. Pearson's linear correlation coefficient, 0.7604, $p < 0.001$.

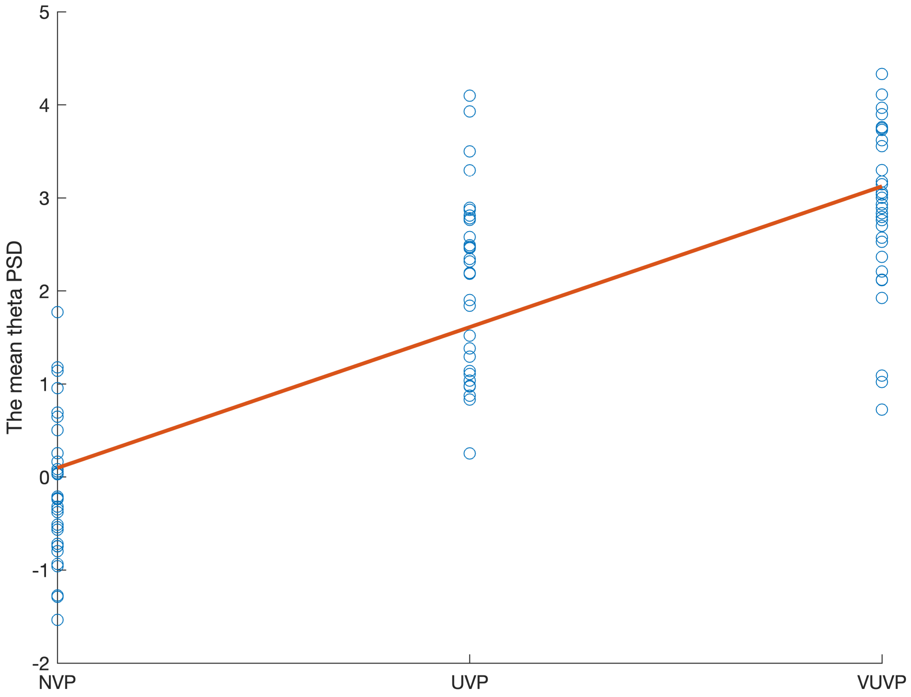

Supplementary Figure 7. The mean theta PSD within the significant time window for the NVP, UVP, and VUVP conditions. Pearson's linear correlation coefficient, 0.7961, $p < 0.001$.

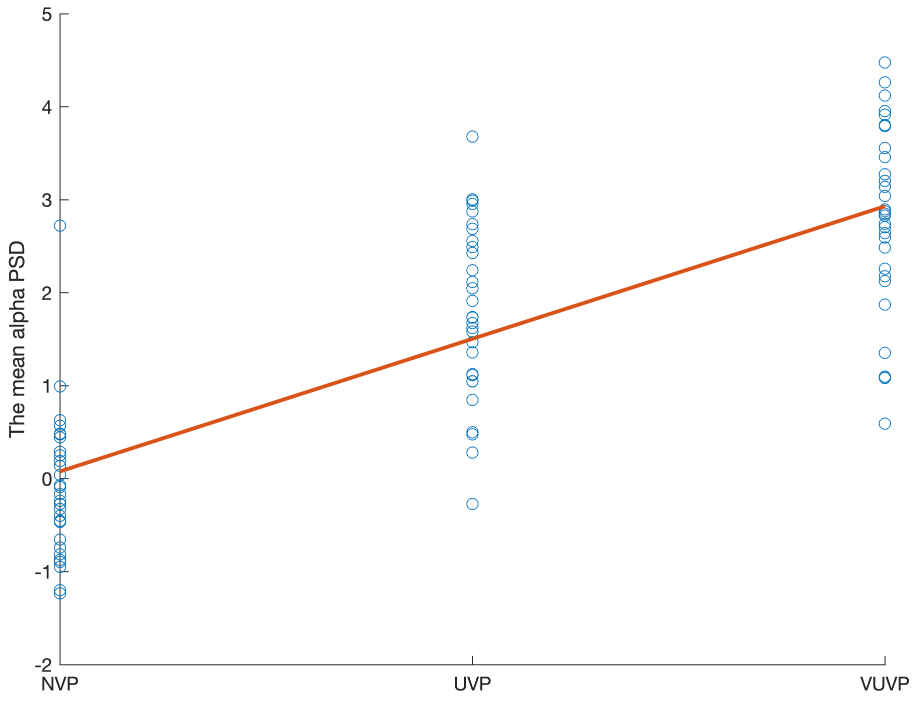

Supplementary Figure 8. The mean alpha PSD within the significant time window for the NVP, UVP, and VUVP conditions. Pearson's linear correlation coefficient, 0.7845, $p < 0.001$.
